# Synthesis and Antibacterial Activity of Novel *N*-Ethylpiperazine-Containing Dihydropyrazolines and Their Chalcone Precursors Against Foodborne and Phytopathogenic Bacteria

**DOI:** 10.3390/molecules31132380

**Published:** 2026-07-06

**Authors:** Meglena I. Kandinska, Peter G. Boyadzhiev, Trayana S. Nedeva, Stanimira T. Ivanova, Viliana D. Miteva, Asya A. Asenova, Vesela V. Lozanova, Valentin S. Lozanov, Iliyana K. Rasheva

**Affiliations:** 1 Faculty of Chemistry and Pharmacy, Sofia University “St. Kliment Ohridski”, 1 James Bourchier Blvd., 1164 Sofia, Bulgaria; pgbhimiq@gmail.com; 2 Faculty of Biology, Sofia University “St. Kliment Ohridski”, 8 Dragan Tsankov Blvd., 1164 Sofia, Bulgaria; nedeva@biofac.uni-sofia.bg (T.S.N.); stanimirti@uni-sofia.bg (S.T.I.); vilianam@uni-sofia.bg (V.D.M.); asjaaa@uni-sofia.bg (A.A.A.); 3 Department of Medical Chemistry and Biochemistry, Medical Faculty, Medical University-Sofia, 2 Zdrave Str., 1431 Sofia, Bulgaria; vlozanova@medfac.mu-sofia.bg (V.V.L.); vlozanov@medfac.mu-sofia.bg (V.S.L.); 4 Centre of Competence “Sustainable Utilization of Bio-resources and Waste of Medicinal and Aromatic Plants for Innovative Bioactive Products” (BIORESOURCES BG), 1000 Sofia, Bulgaria

**Keywords:** dihydropyrazoline, chalcone, *N*-Ethylpiperazine, foodborne pathogens and phytopathogens

## Abstract

Foodborne diseases remain a major global public health concern. According to the World Health Organization (WHO), contaminated food causes nearly 500,000 deaths annually. Consequently, the development of novel strategies to control foodborne pathogens and prevent microorganism-induced plant diseases remains an important research priority, attracting considerable scientific attention worldwide. This study describes the design and synthesis of novel dihydropyrazolines containing an ethylpiperazine moiety as potential antimicrobial agents. The antibacterial activity of the newly synthesized heterocyclic compounds, along with their corresponding chalcone precursors, was evaluated against Gram-positive and Gram-negative foodborne pathogens and phytopathogenic bacteria. The assessment was performed using a two-step protocol comprising an initial qualitative screening of selected bacterial species, followed by a quantitative evaluation of the inhibitory effects of individual compounds and their combinations. Among the tested compounds, pyrazolines **4a** and **4b** and chalcone **3b** exhibited notable strain-dependent antibacterial activity, particularly against phytopathogenic strains of *Pseudomonas syringae*, while compound **4a** demonstrated the highest efficacy against the Gram-negative foodborne pathogens *Escherichia coli*, *Salmonella enterica* and *Listeria monocytogenes*. Thus, the potential of the *N*-ethylpiperazine moiety as a key structural feature contributing to the antimicrobial activity of the studied compounds is revealed.

## 1. Introduction

Foodborne diseases are described as a serious threat to public health and represent a global problem. According to current WHO data, the annual consumption of contaminated food and food products causes nearly 500,000 deaths, 30% of which occur among children under the age of five [1]. *Salmonella* and Shiga-toxin-producing *Escherichia coli* are among the most common foodborne pathogens, while *Listeria monocytogenes* poses a major risk to people in high-risk groups [2]. Plants, including agricultural crops, are also constantly exposed to various pathogens. These phytopathogens can significantly reduce the productivity of major crops worldwide, with annual yield losses ranging from 20 to 40% [3]. At the same time, the ability of certain pathogenic bacteria (phytopathogenic or human-associated) to induce physiological and morphological changes in new hosts (from different kingdoms) has led to the emergence of cross-kingdom pathogenicity [4].

The control of foodborne and plant pathogens affecting plant-based food ingredients is a critical step in the food production chain. This is achieved through the implementation of conventional measures such as monitoring and biosecurity (sanitary conditions, disinfection, pest control, vaccination) through feed and water additives, as well as through preventive safety management systems (good manufacturing practices, good hygiene practices, good agricultural practices, and HACCP) [5,6]. Nevertheless, progress in developing effective control strategies has been slow and is hampered by the adaptation of major food and plant pathogens to various environmental stressors, antimicrobial resistance, heavy metals, biocides, acid tolerance, resistance to Cu^2+^ ions, enhanced colonization/virulence, and biofilm formation, among others [7,8]. In addition, the excessive use of synthetic chemicals with non-specific antimicrobials negatively impacts the environment and human health. Thus, their application in the agricultural sector and food production is discouraged. As a result, researchers worldwide are turning their attention to alternative, environmentally friendly strategies for eliminating the microorganisms responsible for foodborne illnesses and preventing plant diseases caused by microorganisms.

**Figure 1:**
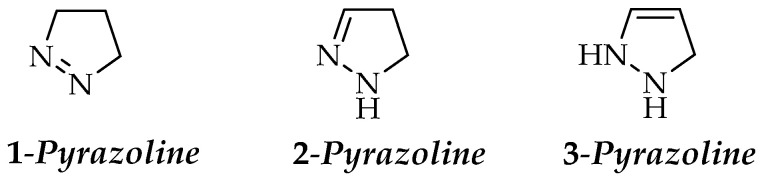
Chemical structures of the isomeric forms of pyrazoline.

Pyrazoline is a dihydro derivative of pyrazole and exists in three isomeric forms—1-pyrazoline, 2-pyrazoline, and 3-pyrazoline (Figure 1)—depending on the position of the endocyclic double bond. In recent years, considerable scientific interest has been directed toward the modification of the 2-pyrazoline ring, owing not only to its stability but also to its occurrence in numerous pharmaceutical agents and the broad spectrum of biological activities exhibited by 2-pyrazoline derivatives [9]. A wide range of 2-pyrazolines has been reported as potential therapeutic agents for Alzheimer’s disease [10] and other age-related disorders [11], as well as anticancer drugs [12]. 2-Pyrazoline derivatives have been identified as novel promising antischistosomal [13] and antioxidant agents [14]. The antimicrobial properties of various dihydropyrazoles [15,16,17,18], including morpholine-containing analogues [19], have also been extensively investigated.

In this study, we report the synthesis of three novel pyrazoline derivatives **4a**–**c** bearing an *N*-ethylpiperazine moiety and evaluation of their antibacterial activity. Considering the significant contribution of the piperazine ring to the biological activity of numerous bioactive compounds [20,21] and given that antibacterial pyrazoline derivatives containing a (4-ethylpiperazin-1-yl)phenyl fragment at the 5-position of the heterocyclic core have not been reported to date, this study aimed to investigate the effect of this previously unexplored structural feature in combination with different 4-substituted phenyl groups at the 3-position of the same heterocycle. To further elucidate the role of the (4-ethylpiperazin-1-yl)phenyl moiety, a pyrazoline derivative **4d** incorporating a 4-piperidinylphenyl fragment at position 5 was also included in the biological evaluation.

In addition, to assess the influence of the pyrazoline ring on the biological properties of the target nitrogen-containing heterocyclic compounds **4a**–**c**, their antibacterial activity was compared with that of the corresponding chalcone precursors **3a**–**c.**

## 2. Results and Discussion

### 2.1. Synthesis

**Scheme 1:**
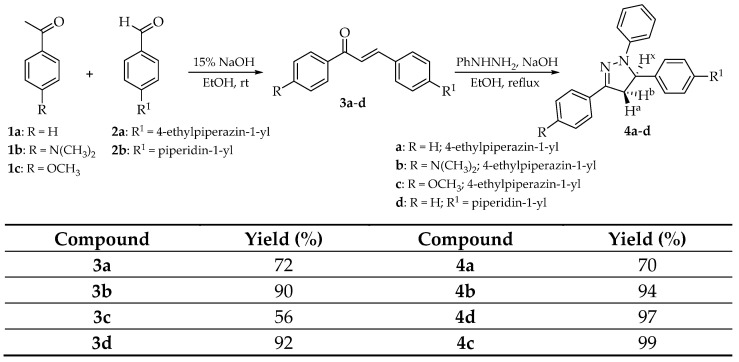
Synthesis and yields of chalcones **3a**–**d** and dihydropyrazolines **4a**–**d**.

The synthetic route for the preparation of the target pyrazoline derivatives **4a**–**d** is outlined in Scheme 1 and comprises a two-step protocol. In the first step, four chalcones **3a**–**d** were synthesized via a base-catalyzed Claisen–Schmidt condensation of acetophenones **1** with 4-substituted benzaldehydes **2** in alcoholic reaction media. Aldehydes **2** were prepared from 4-fluorobenzaldehyde according to a procedure previously described in the literature [22] that provides a convenient synthetic approach for the incorporation of diverse heterocyclic fragments with valuable biological properties, including piperazine and piperidine moieties, into ring B of the corresponding chalcone scaffold. In the second step (Scheme 1), the desired pyrazolines **4a**–**d** were obtained through condensation of chalcones **3a**–**d** with phenylhydrazine and subsequent cyclization under basic conditions. Compounds **3a**–**c** and **4a**–**d** are novel, and their structures were confirmed by ^1^H- and ^13^C-NMR spectroscopy and mass spectral analyses.

The successful cyclization leading to the formation of the pyrazoline ring in compounds of type **4** was confirmed by the presence of characteristic signals corresponding to the diastereotopic methylene protons at position 4 and the methine proton at position 5 of the pyrazoline ring. Three double doublets were observed in the regions of 3.07–3.11 ppm, 3.77–3.78 ppm, and 5.18–5.19 ppm, which were assigned to the H^a^, H^b^, and H^x^ protons (Scheme 1), respectively, with vicinal coupling constants ^3^J_ax_ = 7.0–7.5 Hz and ^3^J_bx_ = 12–12.5 Hz and geminal coupling constant ^2^J_ab_ = 17.0 Hz.

Both types of compounds (**3** and **4**) were isolated as crystalline solids in good to excellent yields without any further purification. In addition, they were characterized by determination of their melting points.

### 2.2. Antimicrobial Activity

This study assessed the antibacterial potential of a series of newly synthesized nitrogen-containing heterocyclic compounds against five bacterial foodborne disease transmitters and five phytopathogens (see Table 1). They encompass type cultures and wild local isolates from infected tomato and pepper plants, the latter kindly provided by Maritsa Vegetable Crops Research Institute, Bulgaria. Among them, sensitive species were selected through a two-step approach. This included an initial qualitative screening of the aforementioned bacteria, followed by quantitative evaluation of the inhibition potential of individual substances or combinations thereof, based on the highest antibacterial activity and species specificity.

The results of the screening procedure are presented in Table 2A,B. As shown, the studied compounds inhibited bacterial growth (9.8 to 23.8 mm zones) in a species- and strain-dependent manner. The strongest effect was observed against *L. monocytogenes* ATCC 13932, followed by *S. enterica* ATCC 14028 and *E. coli* ATCC 8739. Among the model phytopathogens, the *P. syringae* strains (R0 and R1) were susceptible to the inhibitory effect of the studied compounds. Based on these results, the most promising compounds, namely, all investigated pyrazolines **4a**–**d** and a chalcone derivative **3b**, were tested for MIC and MBC against these Gram-negative bacterial cultures.

The MIC and MBC data for the foodborne disease transmitters *L. monocytogenes* ATCC 13932, *S. enterica* ATCC 14028, and *E. coli* ATCC 8739 are shown in Table 3A. They indicate that compounds **3b** and **4a** are effective growth inhibitors of the tested bacterial species. Their MIC values were within the range of 1.25 to 7.50 × 10^2^ µM. The MBC values confirmed this tendency, with the best inhibitory effect demonstrated by compound **4a** (2.50 × 10^2^ µM against *L. monocytogenes* ATCC 13932).

Furthermore, as regards the MIC and MBC of the tested compounds against the phytopathogenic *P. syringae* strains, **3b**, **4a**, and **4b** (Table 3B) demonstrated a potent antimicrobial activity, which was strain dependent and better presented for *Pseudomonas syringae* R1 (MIC = 0.63 × 10^2^ µM and MBC = 1.25 × 10^2^ µM).

The use of newly synthesized compounds with a defined structure and proven targeted microbicidal/microbiostatic effects is a rational approach to studying the impact of chemical substances on the biotic environment. Thus, five newly synthesized compounds were selected and tested for their antibacterial properties and potential application for monitoring cross-pathogenicity and controlling phytopathogens that attack plant-based raw materials. A qualitative screening procedure combined with quantitative analysis of MIC and MBC was applied. The data showed that three of the newly synthesized compounds exhibit potent antibacterial activity. Compounds **4a** and **4b**, which differ solely in the substituent at the 3-position of the pyrazoline ring, together with chalcone **3b**, the corresponding starting material for the synthesis of **4b**, displayed notable, strain-dependent antibacterial effect, especially against the phytopathogenic *Pseudomonas syringae* strains, while pyrazoline **4a** was most efficient against the foodborne disease transmitters *L. monocytogenes*, *E. coli*, and *S. enterica*.

## 3. Materials and Methods

### 3.1. Chemistry

All solvents used in the present study were HPLC grade and are commercial chemical substances. Starting compounds—4-fluorobenzaldehyde, 1-ethylpiperazine, piperidine, acetophenone (**1a**), 1-(4-(dimethylamino)phenyl)ethan-1-one (**1b**), 1-(4-methoxyphenyl)ethan-1-one (**1c**) and phenylhydrazine, are commercially available and were used as supplied. 4-(4-Ethylpiperazin-1-yl)benzaldehyde (**2a**) and 4-(piperidin-1-yl)benzaldehyde (**2b**) were synthesized from 4-fluorobenzaldehyde by nucleophilic substitution with 1-ethylpiperazine or piperidine, respectively, following the procedure reported by Tintory et al. [22], and the structures of the aldehydes **2a** and **2b** were verified through the spectroscopic characterization of the corresponding chalcone products. Compound **3d** is already described in the literature [25]. The melting points (m.p.) of the compounds were determined on Kofler melting point apparatus and are uncorrected. NMR spectra were obtained in CDCl_3_ as a solvent, unless otherwise specified, using a Bruker Avance III 500 HD NMR spectrometer, Bruker BioSpin AG, Fällanden, Switzerland, operating at 500.13 MHz for ^1^H and 125.76 MHz for ^13^C NMR. The chemical shifts are presented in ppm (δ) using tetramethylsilane (TMS) as an internal standard (Supplementary Materials). Liquid chromatography mass spectrometry analysis (LC-MS, Supplementary Materials) was carried out on a Q Ex-active^®^ hybrid quadrupole-Orbitrap^®^ mass spectrometer (Thermo-Scientific Co., Waltham, MA, USA) equipped with a HESI^®^ (heated electrospray ionization) module, TurboFlow^®^ Ultra High-Performance Liquid Chromatography (UHPLC) system (Thermo-Scientific Co., Waltham, MA, USA), and HTC PAL^®^ autosampler (CTC Analytics, Zwingen, Switzerland).

The course of the reactions was monitored by thin-layer chromatography. The studies were carried out with silica gel ALUGRAM^®^ SIL G/UV 25460 Macherey-Nagel, Düren, Germany ready-to-use plates with a layer thickness of 0.2 mm.

*General procedure for the synthesis of chalcones* **3a**–**d**

Equal molar amounts of the starting aldehyde and the corresponding arylmethyl ketone were mixed in alcohol (methanol or ethanol). The reaction mixture was then cooled in an ice bath, and a 20% aqueous sodium hydroxide solution (5-fold molar excess of NaOH) was added. The mixture was stirred at room temperature overnight until complete consumption of the starting carbonyl compounds, as monitored by TLC. The resulting chalcone precipitate was filtered off, washed thoroughly with ethanol, and air-dried.

(*E*)-*3*-(*4*-(*4*-*Ethylpiperazin*-*1*-*yl*)*phenyl*)-*1*-*phenylprop*-*2*-*en*-*1*-*one* (**3a**)

Obtained from **2a** (0.764 g, 3.5 mmol), **1a** (0.421 g, 3.5 mmol) and sodium hydroxide (0.700 g, 17.5 mmol) in 4.0 mL of methanol. Yield = 0.806 g (72%), m.p. = 154–155 °C.

^1^H-NMR δ = 1.14 (t, 3H, H-CH_3_, J = 7.0 Hz), 2.48 (q, 2H, H-CH_2_, J = 7.0 Hz), 2.60 (t, 4H, H-Pip, J = 5.0 Hz), 3.35 (t, 4H, H-Pip, J = 5.0 Hz), 6.90 (d, 2H, H-Ph, J = 8.0 Hz), 7.37 (d, 1H, H-CH, J = 16.0 Hz), 7.49 (t, 2H, H-Ph, J = 8.0 Hz), 7.54–7.57 (m, 3H, H-Ph), 7.77 (d, 1H, H-CH, J = 15.5 Hz), 8.00 (dd, 2H, H-Ph, J = 1.5, 8.0 Hz). ^13^C-NMR δ = 190.69 (C=O), 152.77, 145.22, 138.85, 132.33, 130.17 (2C), 128.51 (2C), 128.36 (2C), 125.23, 118.38, 114.75 (2C), 52.57 (2C), 52.36, 47.77 (2C), 12.00. LC-MS Chemical Formula: C_21_H_24_N_2_O, Exact Mass [M+H]^+^: 321.19614, Found: [M+H]^+^: 321.19586.

(*E*)-*1*-(*4*-(*Dimethylamino*)*phenyl*)-*3*-(*4*-(*4*-*ethylpiperazin*-*1*-*yl*)*phenyl*)*prop*-*2*-*en*-*1*-*one* (**3b**)

Obtained from **2a** (1.42 g, 6.5 mmol), **1b** (1.06 g, 6.5 mmol) and sodium hydroxide (0.260 g, 6.5 mmol) in 7.5 mL of ethanol. Yield = 2.13 g (90%), m.p. = 190–192 °C.

^1^H-NMR δ = 1.07 (t, 3H, H-CH_3_, J = 7.5 Hz), 2.40 (q, 2H, H-CH_2_, J = 7.5 Hz), 2.53 (t, 4H, H-Pip, J = 5.0 Hz), 3.00 (s, 6H, H-NCH_3_), 3.25 (t, 4H, H-Pip, J = 5.0 Hz), 6.63 (d, 2H, H-Ph, J = 8.5 Hz), 6.83 (d, 2H, H-Ph, J = 8.5 Hz), 7.37 (d, 1H, H-CH, J = 15.5 Hz), 7.48 (d, 2H, H-Ph, J = 8.5 Hz), 7.87 (d, 1H, H-CH, J = 15.5 Hz), 7.93 (d, 2H, H-Ph, J = 8.5 Hz). ^13^C-NMR δ = 188.02 (C=O), 153.21, 152.37, 142.82, 130.64 (2C), 129.76 (2C), 126.52, 126.06, 118.60, 114.93 (2C), 110.82 (2C), 52.63 (2C), 52.37, 47.99 (2C), 40.08 (2C), 12.01. LC-MS Chemical Formula: C_23_H_29_N_3_O, Exact Mass [M+H]^+^: 364.23833, Found: [M+H]^+^: 364.23853.

(*E*)-*3*-(*4*-(*4*-*Ethylpiperazin*-*1*-*yl*)*phenyl*)-*1*-(*4*-*methoxyphenyl*)*prop*-*2*-*en*-*1*-*one* (**3c**)

Obtained from **2a** (0.764 g, 3.5 mmol), **1c** (0.525 g, 3.5 mmol) and sodium hydroxide (0.140 g, 3.5 mmol) in 4.0 mL of ethanol. Yield = 0.692 g (56%), m.p. = 143–144 °C.

^1^H-NMR δ = 1.07 (t, 3H, H-CH_3_, J = 7.0 Hz), 2.41 (q, 2H, H-CH_2_, J = 7.0 Hz), 2.53 (t, 4H, H-Pip, J = 5.0 Hz), 3.26 (t, 4H, H-Pip, J = 5.0 Hz), 3.81 (s, 3H, H-CH_3_), 6.83 (d, 2H, H-Ph, J = 9.0 Hz), 6.90 (d, 2H, H-Ph, J = 9.0 Hz), 7.32 (d, 1H, H-CH, J = 15.5 Hz), 7.48 (d, 2H, H-Ph, J = 8.5 Hz), 7.70 (d, 1H, H-CH, J = 15.5 Hz), 7.96 (d, 2H, H-Ph, J = 9.0 Hz). ^13^C-NMR δ = 189.89 (C=O), 163.11, 152.63, 144.31, 131.66, 130.62 (2C), 130.02 (2C), 125.49, 118.17, 114.81 (2C), 113.73 (2C), 55.48, 52.59 (2C), 52.36, 47.84 (2C), 12.01. LC-MS Chemical Formula: C_22_H_26_N_2_O_2_, Exact Mass [M+H]^+^: 351.20670, Found: [M+H]^+^: 351.20648.

(*E*)-*1*-*Phenyl*-*3*-(*4*-(*piperidin*-*1*-*yl*)*phenyl*)*prop*-*2*-*en*-*1*-*one* (**3d**)

Obtained from **2b** (1.10 g, 5.8 mmol), **1a** (0.698 g, 0.685 mL, 5.8 mmol) and sodium hydroxide (1.16 g, 29.0 mmol) in 6.5 mL of methanol. Yield = 1.56 g (92%).

^1^H-NMR (CDCl_3_) δ 1.55–1.64 (m, 6H, H-Piperidine), 3.24 (t, 4H, H-Piperidine, J = 5.0 Hz), 6.82 (d, 2H, H-Ph, J = 8.5 Hz), 7.27 (d, 1H, H-CH, J = 15.5 Hz), 7.41 (t, 2H, H-Ph, J = 7.5 Hz), 7.46–7.50 (m, 3H, H-Ph), 7.70 (d, 1H, H-CH, J = 15.5 Hz), 7.93 (d, 2H, H-Ph, J = 7.5 Hz).

*General procedure for the synthesis of dihydropyrazolines* **4a**–**d**

The corresponding chalcone (0.5 mmol), phenylhydrazine (1.0 mmol), solid sodium hydroxide (1.25 mmol) and ethanol (4 mL) were mixed into a round bottom flask, fitted with a condenser. The reaction mixture was refluxed for 3–4 h until the complete consumption of the starting chalcone, as monitored by TLC. The resulting precipitate of dihydropyrazoline was filtered off, washed thoroughly with ethanol and air-dried.

*1*-(*4*-(*1*,*3*-*Diphenyl*-*4*,*5*-*dihydro*-1*H*-*pyrazol*-*5*-*yl*)*phenyl*)-*4*-*ethylpiperazine* (**4a**)

Obtained from **3a** (0.160 g), phenylhydrazine (0.108 g) and solid sodium hydroxide (0.050 g). Yield = 0.144 g (70%), m.p. = 155–156 °C.

^1^H-NMR δ = 1.12 (t, 3H, H-CH_3_, J = 7.0 Hz), 2.46 (q, 2H, H-CH_2_, J = 7.0 Hz), 2.58 (t, 4H, H-Pip, J = 4.5 Hz), 3.11 (dd, 1H, H^a^-CH_2_, ^3^J_ax_ = 7.0 Hz, ^2^J_ab_ = 17.0 Hz), 3.19 (t, 4H, H-Pip, J = 4.5Hz), 3.78 (dd, 1H, H^b^-CH_2_, ^3^J_bx_ = 12.5 Hz, ^2^J_ab_ = 17.0 Hz), 5.19 (dd, 1H, H^x^-CH, ^3^J_ax_ = 7.5 Hz, ^3^J_bx_ = 12.5 Hz), 6.76 (t, 1H, H-Ph, J = 7.5 Hz), 6.87 (d, 2H, H-Ph, J = 9.0 Hz), 7.08 (d, 2H, H-Ph, J = 8.0 Hz), 7.17 (dt, 2H, H-Ph, J = 1.5, 7.5 Hz), 7.20 (d, 2H, H-Ph, J = 8.5 Hz), 7.31 (t, 1H, H-Ph, J = 7.5 Hz), 7.37 (dt, 2H, H-Ph, J = 1.5, 7.5 Hz), 7.71 (d, 2H, H-Ph, J = 7.5 Hz). ^13^C-NMR δ = 150.82, 146.73, 145.04, 133.36, 132.92, 128.85 (2C), 128.52 (2C), 128.48, 126.75 (2C), 125.71 (2C), 118.96, 116.31 (2C), 113.44 (2C), 64.13, 52.86 (2C), 52.37, 48.92 (2C), 43.57, 11.99. LC-MS Chemical Formula: C_27_H_30_N_4_, Exact Mass [M+H]^+^: 411.25432, Found: [M+H]^+^: 411.25452.

*4*-(*5*-(*4*-(*4*-*Ethylpiperazin*-*1*-*yl*)*phenyl*)-*1*-*phenyl*-*4*,*5*-*dihydro*-*1H*-*pyrazol*-*3*-*yl*)-*N*,*N*-*dimethylaniline* (**4b**)

Obtained from **3b** (0.182 g), phenylhydrazine (0.108 g) and solid sodium hydroxide (0.050 g). Yield = 0.214 g (94%), m.p. = 217–219 °C.

^1^H-NMR δ = 1.12 (t, 3H, H-CH_3_, J = 7.0 Hz), 2.46 (q, 2H, H-CH_2_, J = 7.0 Hz), 2.58 (t, 4H, H-Pip, J = 5.0 Hz), 2.99 (s, 6H, H-CH_3_ (NCH_3_)), 3.07 (dd, 1H, H^a^-CH_2_, ^3^J_ax_ = 7.5 Hz, ^2^J_ab_ = 17.0 Hz), 3.19 (dd, 4H, H-Pip, J = 4.5, 6.0 Hz), 3.74 (dd, 1H, H^b^-CH_2_, ^3^J_bx_ = 12.0 Hz, ^2^J_ab_ = 17.0 Hz), 5.19 (dd, 1H, H^x^-CH, ^3^J_ax_ = 7.5 Hz, ^3^J_bx_ = 12.0 Hz), 6.69–6.73 (m, 3H, H-Ph), 6.87 (d, 2H, H-Ph, J = 9.0 Hz), 7.05 (d, 2H, H-Ph, J = 7.5 Hz), 7.14 (dd, 2H, H-Ph, J = 7.0, 8.5 Hz), 7.21 (d, 2H, H-Ph, J = 8.5 Hz), 7.59 (d, 2H, H-Ph, J = 9.0 Hz). ^13^C-NMR(CDCl_3_) δ 150.70, 150.50, 147.68, 145.70, 133.93, 128.77 (2C), 127.03 (2C), 126.81 (2C), 120.96, 118.28, 116.30 (2C), 113.21 (2C), 111.98 (2C), 63.98, 52.88 (2C), 52.37, 48.98 (2C), 43.93, 40.37 (2C), 11.99. LC-MS Chemical Formula: C_29_H_35_N_5_, Exact Mass [M+H]+: 453.28869, Found: [M+H]^+^: 453.28922.

*1*-*Ethyl*-*4*-(*4*-(*3*-(*4*-*methoxyphenyl*)-*1*-*phenyl*-*4*,*5*-*dihydro*-*1H*-*pyrazol*-*5*-*yl*)*phenyl*)*piperazine* (**4c**)

Obtained from **3c** (0.175 g), phenylhydrazine (0.108 g) and solid sodium hydroxide (0.050 g). Yield = 0.214 g (97%), m.p. = 172–174 °C.

^1^H-NMR δ = 1.04 (t, 3H, H-CH_3_, J = 7.5 Hz), 2.38 (q, 2H, H-CH_2_, J = 7.5 Hz), 2.55 (t, 4H, H-Pip, J = 5.0 Hz), 3.00 (dd, 1H, H^a^-CH_2_, ^3^J_ax_ = 7.5 Hz, ^2^J_ab_ = 17.0 Hz), 3.12 (dd, 4H, H-Pip, J = 4.5, 6.0 Hz), 3.67 (dd, 1H, H^b^-CH_2_, ^3^J_bx_ = 12.5 Hz, ^2^J_ab_ = 17.0 Hz), 3.75 (s, 3H, H-OCH_3_), 5.06 (dd, 1H, H^x^-CH, ^3^J_ax_ = 7.5 Hz, ^3^J_bx_ = 12.5 Hz), 6.67 (t, 1H, H-Ph, J = 7.0 Hz), 6.79 (d, 2H, H-Ph, J = 9.0 Hz), 6.82 (d, 2H, H-Ph, J = 9.0 Hz), 6.98 (d, 2H, H-Ph, J = 9.0 Hz), 7.08 (dd, 2H, H-Ph, J = 7.0, 9.0 Hz), 7.12 (d, 2H, H-Ph, J = 8.5 Hz), 7.57 (d, 2H, H-Ph, J = 8.5 Hz). ^13^C-NMR δ = 160.05, 150.58, 146.82, 145.35, 128.83 (2C), 127.19 (2C), 126.77 (2C), 125.71, 118.68, 116.31 (2C), 113.99 (2C), 113.33 (2C), 64.12, 55.35, 52.86 (2C), 52.37, 48.94 (2C), 43.82, 11.98. LC-MS Chemical Formula: C_28_H_32_N_4_O, Exact Mass [M+H]^+^: 441.26488, Found: [M+H]^+^: 441.26517.

*1*-(*4*-(*1*,*3*-*Diphenyl*-*4*,*5*-*dihydro*-*1H*-*pyrazol*-*5*-*yl*)*phenyl*)*piperidine* (**4d**)

Obtained from **3d** (0.145 g), phenylhydrazine (0.108 g) and solid sodium hydroxide (0.050 g). Yield = 0.188 g (99%), 186–188 °C.

^1^H-NMR δ = 1.53 (m, 2H, H-Piperidine), 1.66–1.70 (m, 4H, H-Piperidine), 3.09–3.14 (m, 5H, H-Piperidine and H^a^-CH_2_), 3.77 (dd, 1H, H^b^-CH_2_, ^3^J_bx_ = 12.0 Hz, ^2^J_ab_ = 17.0 Hz), 5.18 (dd, 1H, H^x^-CH, ^3^J_ax_ = 7.0 Hz, ^3^J_bx_ = 12.0 Hz), 6.76 (t, 1H, H-Ph, J = 7.0 Hz), 6.87 (d, 2H, H-Ph, J = 9.0 Hz), 7.08 (dd, 2H, H-Ph, J = 1.5; 9.0 Hz), 7.15–7.19 (m, 4H, H-Ph), 7.29–7.32 (m, 2H, H-Ph), 7.37 (dt, 2H, H-Ph, J = 1.5; 7.0 Hz), 7.71 (dd, 2H, H-Ph, J = 1.5; 9.0 Hz). ^13^C-NMR δ = 151.54, 146.74, 145.06, 132.97, 132.82, 128.83 (2C), 128.51 (2C), 128.45, 126.62 (2C), 125.71 (2C), 118.93, 116.74 (2C), 113.46 (2C), 64.21, 50.42 (2C), 43.59, 25.87 (2C), 24.27. LC-MS Chemical Formula: C_26_H_27_N_3_, Exact Mass [M+H]^+^: 382.22777, Found: [M+H]^+^: 382.22784.

### 3.2. Antimicrobial Activity

#### 3.2.1. Screening for Antimicrobial Activity

The newly synthesized nitrogen-containing heterocyclic compounds were screened for antimicrobial activity against Gram-positive and Gram-negative bacteria responsible for transmission of foodborne illnesses and plant diseases (Table 1) using the agar diffusion (well) method [26] and results interpreted in accordance with CLSI standards [27]. The foodborne diseases’ transmitters were grown on Tryptic Soy Agar (TSA) (HiMedia, Mumbai, Maharashtra, India) at 37 °C for 24 h (except *B. cereus* ATCC 11778—at 28 °C for 24 h), and the phytopathogens on Potato Dextrose Agar (PDA) (HiMedia, Mumbai, Maharashtra, India) at 28 °C for 24 h. Microbial suspensions were prepared using Ringer’s solution (Merck Millipore, Darmstadt, Germany) until the turbidity reached a level equivalent to a 0.5 McFarland standard (ca. 1 × 10^8^ CFU/mL). A total of 100 µL from each suspension was inoculated in the relevant agar plates, melted and cooled to 45–50 °C. Following solidification, wells were drilled in the inoculated agar plates using a sterile cork-borer (6 mm in diameter). Solutions of the tested newly synthesized nitrogen-containing heterocyclic compounds were prepared in dimethyl sulfoxide (DMSO) (Merck Millipore, Darmstadt, Germany) at 1.00 mM and 0.50 mM. Each well was loaded with 100 µL of the corresponding test solution, and the agar plates were incubated at 4 °C for 30 min, allowing the test compound to diffuse in the medium. The agar plates were then cultured at the relevant temperature (see above) for 18–20 h. After the incubation period, the antimicrobial effect was verified by measuring the size of the inhibition zones (incl. the well width). The results were compared with those of a positive control, commercial antibiotics chloramphenicol (30 µg/disc) and amoxicillin (30 µg/disc) (HiMedia, Mumbai, Maharashtra, India). DMSO was used as a negative control.

#### 3.2.2. Determination of Minimum Inhibitory and Minimum Bactericidal Concentrations

The Minimum Inhibitory Concentration (MIC) was determined by real-time monitoring of microbial growth. A microplate reader (BMG Labtech, Ortenberg, Germany) with an automated data acquisition and analysis module was used for measuring OD_600_. The microplate dilution method was applied using 48-well plates with a working volume of 300 µL. The tested compounds were used to prepare two-fold dilutions, and 150 µL of each was transferred into the microplate, followed by the addition of 150 µL stock solution of the tested microbial strains (0.5 McFarland standard in the relevant media). The tested compounds were at final concentrations of 0.75 to 0.0156 mM. As a positive control for growth, 150 µL of the test strain stock solution was mixed with 150 µL of the relevant medium. The negative control (assay background) contained the tested compounds and the corresponding nutrient medium. The microplates were incubated for 24 h at the relevant temperature for the foodborne disease transmitters and the phytopathogens. The MIC values were determined in accordance with CLSI standards [28].

The Minimum Bactericidal Concentration (MBC) was determined by subculturing the broth dilutions of the microplates, corresponding to and above the MIC. The broth cultures were serially tenfold diluted and plated dropwise on the relevant solid nutrient medium. The MBC was defined as the lowest concentration of the tested compounds showing no visible microbial growth.

All assays were performed in triplicate and repeated three times. Data were processed using the MS Excel inbuilt functions, and results are presented as means ± standard deviations (*n* = 3).

## 4. Conclusions

In this article, we report the synthesis and evaluation of the antibacterial activity of three novel pyrazoline derivatives, **4a**–**c**, bearing an *N*-ethylpiperazine moiety against foodborne and phytopathogens. Their antibacterial properties were compared with those of the corresponding precursors—chalcones **3a**–**c**, which have not previously been described in the literature. Our results indicate that 2-pyrazolines, containing *N*-ethylpiperazine fragment at the 5-position of the main heterocyclic core, namely, **4a** and **4b**, possess notable inhibitory potential with MICs within the 1.25–7.5 × 10^2^ µM range and may serve as promising lead compounds for the design and development of novel derivatives with enhanced antimicrobial activity. A comparison of the biological activities of products **4** and chalcones **3** (Table 2A,B and Table 3A,B) suggests that the transformation of the latter into heterocyclic derivatives **4**, involving the formation of a five-membered dihydropyrazoline ring, plays a significant role in enhancing the antibacterial activity of these compounds. Furthermore, an analysis of the antibacterial properties of the *N*-ethylpiperazine-containing pyrazoline **4a** and its piperidine analogue **4d** (Table 2A,B and Table 3A,B) indicates that, at this stage of our investigations, the *N*-ethylpiperazine moiety may be considered a key structural feature contributing to the antimicrobial activity exhibited by the studied compounds. However, further analyses are needed to elucidate the cytotoxicity profile of the studied pyrazolines to fully reveal their biopotential as antimicrobial leads.

## Figures and Tables

**Table 1:** Gram-positive and Gram-negative bacteria responsible for food-born illnesses transmission and plant diseases.

Foodborne Disease Transmitters	Phytopathogenic Bacteria
*Escherichia coli* ATCC 8739	*Xanthomonas vesicatoria* [23]
*Salmonella enterica* ATCC 14028	*Xanthomonas euvesicatoria* [24]
*Bacillus cereus* ATCC 11778	*Xanthomonas gardneri*
*Staphylococcus aureus* ATCC 6538	*Pseudomonas syringae* R0 [23]
*Listeria monocytogenes* ATCC 13932	*Pseudomonas syringae* R1 [23]

**Table 2:** (**A**) *In vitro* antibacterial activities of compounds **3a**–**c** and **4a**–**d** against foodborne disease transmitters. CLPH—chloramphenicol. (**B**) *In vitro* antibacterial activities of compounds **3b** and **4a–d** against phytopathogenic bacteria. AMX—amoxicillin.

(A)						
Compound		Inhibition zone (mm)				
	mM	*S. aureus* ATCC 6538	*B. cereus* ATCC 11778	*L. monocytogenes* ATCC 13932	*E. coli* ATCC 8739	*S. enterica* ATCC 14028
**3a**	1.00	0	0	13.5 ± 0.5	0	13.2 ± 0.3
	0.50	0	0	0	0	0
**3b**	1.00	0	0	0	16.2 ± 0.4	18.0 ± 0.5
	0.50	0	0	0	0	11.8 ± 0.2
**3c**	1.00	0	0	15.4 ± 0.6	12.8 ± 0.2	15.3 ± 0.6
	0.50	0	0	0	0	0
**4a**	1.00	12.1 ± 0.1	0	19.2 ± 0.6	14.1 ± 0.4	17.4 ± 0.5
	0.50	0	0	12.5 ± 0.2	0	16.0 ± 0.4
**4b**	1.00	0	0	0	14.7 ± 0.6	14.6 ± 0.5
	0.50	0	0	0	0	0
**4c**	1.00	11.8 ± 0.1	0	15.0 ± 0.5	13.4 ± 0.3	12.3 ± 0.2
	0.50	0	0	13.2 ± 0.3	0	0
**4d**	1.00	0	0	23.8 ± 1.1	15.1 ± 0.6	16.1 ± 0.4
	0.50	0	0	0	0	10.8 ± 0.1
CLPH		19.8 ± 0.3	24.3 ± 0.6	31.0 ± 0.8	31.46 ± 0.9	15.9 ± 0.3
DMSO	50%	0	0	0	0	0
(B)						
Compound		Inhibition zone (mm)				
	mM	*X. gardneri*	*X. vesicatoria*	*X. euvesicatoria*	*P. syringae* R0	*P. syringae* R1
**3b**	1.00	0	0	0	13.2 ± 0.9	15.1 ± 1.4
	0.50	0	0	0	12.0 ± 1.0	0
**4a**	1.00	0	0	0	13.9 ± 1.2	13.2 ± 1.2
	0.50	0	0	0	12.4 ± 1.1	0
**4b**	1.00	0	0	0	11.6 ± 1.3	16.8 ± 1.6
	0.50	0	0	0	0	0
**4c**	1.00	0	0	0	9.8 ± 0.1	0
	0.50	0	0	0	0	0
**4d**	1.00	0	0	0	13.1 ± 1.1	0
	0.50	0	0	0	0	0
AMX		23.0 ± 0.3	29.0 ± 0.8	25.1 ± 0.6	25.1 ± 0.6	26.1 ± 0.9
DMSO	50%	0	0	0	0	0

*All results are presented as means ± standard deviations of three independent replicates (n = 3).*

**Table 3:** (**A**) Minimal Inhibitory Concentrations (MIC) and Minimal Bactericidal Concentrations (MBC) of compounds **3b** and **4a**–**d** against *L. monocytogenes*, *E. coli*, and *S. enterica* species. (**B**) Minimal Inhibitory Concentrations (MIC) and Minimal Bactericidal Concentrations (MBC) of compounds **3b** and **4a**–**d** against *P. syringae* strains.

(A)													
	Strain	*L. monocytogenes* ATCC 13932 (µM × 10^2^)				*E. coli* ATCC 8739 (µM × 10^2^)				*S. enterica* ATCC 14028 (µM × 10^2^)			
Compound		MIC		MBC		MIC		MBC		MIC		MBC	
**3b**		>10.0		>10.0		3.75 ± 0.42		7.50 ± 0.94		7.50 ± 0.43		10.0 ± 1.4	
**4a**		1.25 ± 0.33		2.50 ± 0.42		2.50 ± 0.22		5.00 ± 0.83		3.75 ± 0.32		7.5 ± 0.67	
**4b**		>10.0		>10.0		>10.0		>10.0		>10.0		>10.0	
**4c**		10.0 ± 1.3		>10.0		>10.0		>10.0		>10.0		>10.0	
**4d**		10.0 ± 1.2		>10.0		10.0 ± 1.5		>10.0		10.0 ± 1.5		10.0 ± 1.3	
(B)													
	Strain	*P. syringae* R0 (µM × 10^2^)						*P. syringae* R1 (µM × 10^2^)					
Compound		MIC			MBC			MIC			MBC		
**3b**		1.25 ± 0.31			2.5 ± 0.44			**3b**			1.25 ± 0.31		
**4a**		1.25 ± 0.29			2.5 ± 0.46			**4a**			1.25 ± 0.29		
**4b**		1.25 ± 0.33			2.5 ± 0.49			**4b**			1.25 ± 0.33		
**4c**		>10.0			>10.0			**4c**			>10.0		
**4d**		>10.0			>10.0			**4d**			>10.0		

*All results are presented as means ± standard deviations of three independent replicates (n = 3).*

## Data Availability

The original contributions presented in this study are included in the article/Supplementary Material. Further inquiries can be directed to the corresponding author.

## Supplementary Materials

The following supporting information can be downloaded at: https://www.mdpi.com/article/10.3390/molecules31132380/s1, Figure S1: ^1^H-NMR Spectrum (CDCl_3_, 500 MHz) (*E*)-3-(4-(4-Ethylpiperazin-1-yl)phenyl)-1-phenylprop-2-en-1-one (**3a**); Figure S2: ^13^C-NMR Spectrum (CDCl_3_, 125.76 MHz) (*E*)-3-(4-(4-Ethylpiperazin-1-yl)phenyl)-1-phenylprop-2-en-1-one (**3a**); Figure S3: LC-MS of (*E*)-3-(4-(4-Ethylpiperazin-1-yl)phenyl)-1-phenylprop-2-en-1-one (**3a**); Figure S4: ^1^H-NMR Spectrum (CDCl_3_, 500 MHz) of (*E*)-1-(4-(Dimethylamino)phenyl)-3-(4-(4-ethylpiperazin-1-yl)phenyl)prop-2-en-1-one (**3b**); Figure S5: ^13^C-NMR Spectrum (CDCl_3_, 125.76 MHz) of (*E*)-1-(4-(Dimethylamino)phenyl)-3-(4-(4-ethylpiperazin-1-yl)phenyl)prop-2-en-1-one (**3b**); Figure S6: LC-MS of (*E*)-1-(4-(Dimethylamino)phenyl)-3-(4-(4-ethylpiperazin-1-yl)phenyl)prop-2-en-1-one (**3b**); Figure S7: ^1^H-NMR Spectrum (CDCl_3_, 500 MHz) of (*E*)-3-(4-(4-Ethylpiperazin-1-yl)phenyl)-1-(4-methoxyphenyl)prop-2-en-1-one (**3c**); Figure S8: ^13^C-NMR Spectrum (CDCl_3_, 125.76 MHz) of (*E*)-3-(4-(4-Ethylpiperazin-1-yl)phenyl)-1-(4-methoxyphenyl)prop-2-en-1-one (**3c**); Figure S9: LC-MS of (*E*)-3-(4-(4-Ethylpiperazin-1-yl)phenyl)-1-(4-methoxyphenyl)prop-2-en-1-one (**3c**); Figure S10: ^1^H-NMR Spectrum (CDCl_3_, 500 MHz) of 1-(4-(1,3-Diphenyl-4,5-dihydro-1*H*-pyrazol-5-yl)phenyl)-4-ethylpiperazine (**4a**); Figure S11: ^13^C-NMR Spectrum (CDCl_3_, 125.76 MHz) of 1-(4-(1,3-Diphenyl-4,5-dihydro-1*H*-pyrazol-5-yl)phenyl)-4-ethylpiperazine (**4a**); Figure S12: LC-MS of 1-(4-(1,3-Diphenyl-4,5-dihydro-1*H*-pyrazol-5-yl)phenyl)-4-ethylpiperazine (**4a**); Figure S13: ^1^H-NMR Spectrum (CDCl_3_, 500 MHz) of 4-(5-(4-(4-Ethylpiperazin-1-yl)phenyl)-1-phenyl-4,5-dihydro-1*H*-pyrazol-3-yl)-*N*,*N*-dimethylaniline (**4b**); Figure S14: ^13^C-NMR Spectrum (CDCl_3_, 125.76 MHz) of 4-(5-(4-(4-Ethylpiperazin-1-yl)phenyl)-1-phenyl-4,5-dihydro-1*H*-pyrazol-3-yl)-*N*,*N*-dimethylaniline (**4b**); Figure S15: LC-MS of 4-(5-(4-(4-Ethylpiperazin-1-yl)phenyl)-1-phenyl-4,5-dihydro-1*H*-pyrazol-3-yl)-*N*,*N*-dimethylaniline (**4b**); Figure S16: ^1^H-NMR Spectrum (CDCl_3_, 500 MHz) of 1-Ethyl-4-(4-(3-(4-methoxyphenyl)-1-phenyl-4,5-dihydro-1*H*-pyrazol-5-yl)phenyl)piperazine (**4c**); Figure S17: ^13^C-NMR Spectrum (CDCl_3_, 125.76 MHz) of 1-Ethyl-4-(4-(3-(4-methoxyphenyl)-1-phenyl-4,5-dihydro-1*H*-pyrazol-5-yl)phenyl)piperazine (**4c**); Figure S18: LC-MS of 1-Ethyl-4-(4-(3-(4-methoxyphenyl)-1-phenyl-4,5-dihydro-1*H*-pyrazol-5-yl)phenyl)piperazine (**4c**); Figure S19: ^1^H-NMR Spectrum (CDCl_3_, 500 MHz) of 1-(4-(1,3-Diphenyl-4,5-dihydro-1*H*-pyrazol-5-yl)phenyl)piperidine (**4d**); Figure S20: ^13^C-NMR Spectrum (CDCl_3_, 125.76 MHz) of 1-(4-(1,3-Diphenyl-4,5-dihydro-1*H*-pyrazol-5-yl)phenyl)piperidine (**4d**); Figure S21: LC-MS of 1-(4-(1,3-Diphenyl-4,5-dihydro-1*H*-pyrazol-5-yl)phenyl)piperidine (**4d**).
