# Supplementary material for: Synthesis and Antibacterial Activity of Novel N-Ethylpiperazine-Containing Dihydropyrazolines and Their Chalcone Precursors Against Foodborne and Phytopathogenic Bacteria
Source: Molecules. 2026 Jul 6;31(13):2380. doi: 10.3390/molecules31132380 (PMC13362652; doi:10.3390/molecules31132380)

Synthesis and Antibacterial Activity of Novel *N*-Ethylpiperazine-Containing Dihydropyrazolines and Their Chalcone Precursors against Foodborne and Phytopathogenic Bacteria

Meglena I. Kandinska[1*], Peter G. Boyadzhiev[1], Trayana S. Nedeva[2], Stanimira T. Ivanova[2], Viliana D. Miteva[2], Asya A. Asenova[2], Vesela V. Lozanova[3], Valentin S. Lozanov[3], Iliyana K. Rasheva[2,4*]

[1]Sofia University "St. Kliment Ohridski", Faculty of Chemistry and Pharmacy, 1 James Bourchier Blvd., 1164 Sofia, Bulgaria; ohmk@chem.uni-sofia.bg, pgbhimiq@gmail.com
[2]Sofia University "St. Kliment Ohridski", Faculty of Biology, 8 Dragan Tsankov Blvd., 1164 Sofia, Bulgaria; nedeva@biofac.uni-sofia.bg, stanimirti@uni-sofia.bg, vilianam@uni-sofia.bg, asjaaa@uni-sofia.bg, i_rasheva@biofac.uni-sofia.bg
[3]Medical University-Sofia, Department of Medical Chemistry and Biochemistry, Medical Faculty, 2 Zdrave Str., 1431 Sofia, Bulgaria; vlozanov@medfac.mu-sofia.bg, vlozanova@medfac.mu-sofia.bg
[4]Centre of Competence "Sustainable Utilization of Bio-resources and Waste of Medicinal and Aromatic Plants for Innovative Bioactive Products" (BIORESOURCES BG), 1000 Sofia, Bulgaria

*Correspondence: ohmk@chem.uni-sofia.bg; +359 887 195554 and i_rasheva@biofac.uni-sofia.bg; +359 882 216969

Content

1. NMR Spectra (Figures S1 and S2) and of LC-MS Spectrum (Figure S3) of **3a**
2. NMR Spectra (Figures S4 and S5) and of LC-MS Spectrum (Figure S6) of **3b**
3. NMR Spectra (Figures S7 and S8) and of LC-MS Spectrum (Figure S9) of **3c**
4. NMR Spectra (Figures S10 and S11) and of LC-MS Spectrum (Figure S12) of **4a**
5. NMR Spectra (Figures S13 and S4) and of LC-MS Spectrum (Figure S15) of **4b**
6. NMR Spectra (Figures S16 and S17) and of LC-MS Spectrum (Figure S18) of **4c**
7. NMR Spectra (Figures S19 and S20) and of LC-MS Spectrum (Figure S21) of **4d**

Figure S1. ^{1}H-NMR Spectrum ($CDCl_3$, 500 MHz) (*E*)-3-(4-(4-Ethylpiperazin-1-yl)phenyl)-1-phenylprop-2-en-1-one (**3a**).

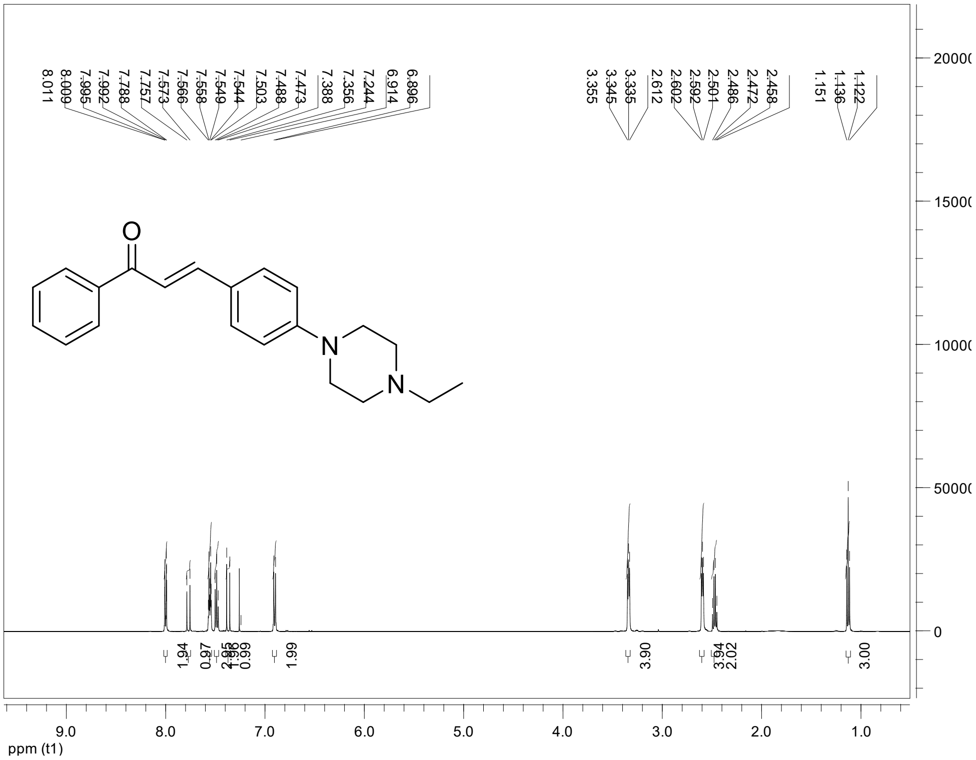

Figure S2. ^{13}C-NMR Spectrum ($CDCl_3$, 125.76 MHz) (*E*)-3-(4-(4-Ethylpiperazin-1-yl)phenyl)-1-phenylprop-2-en-1-one (**3a**).

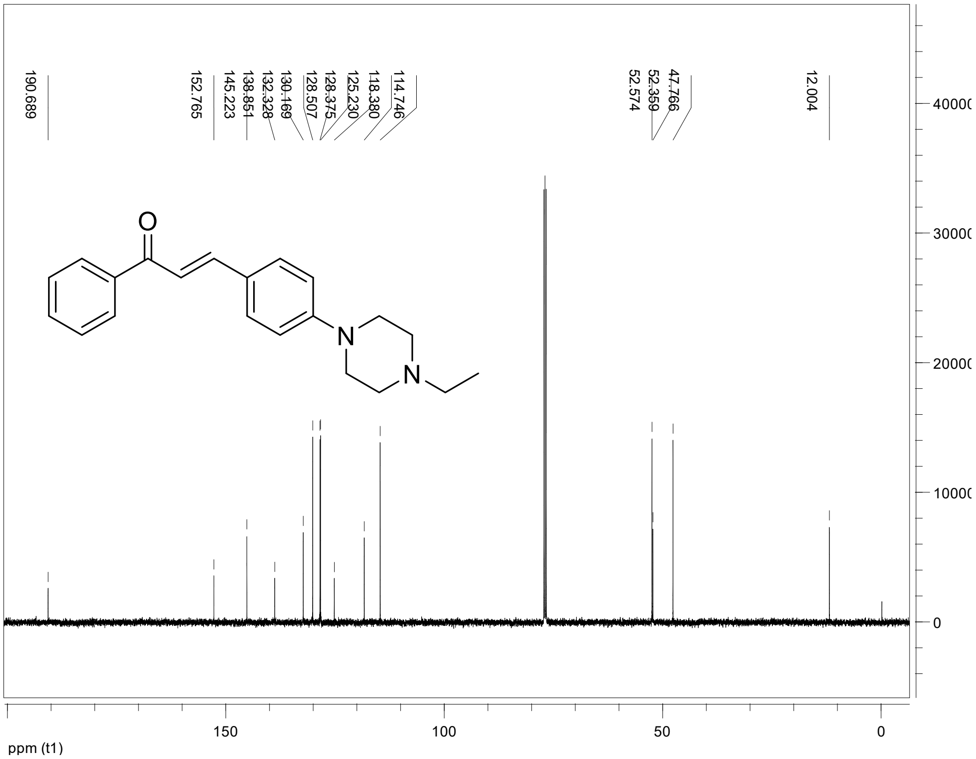

Figure S3. LC-MS of (*E*)-3-(4-(4-Ethylpiperazin-1-yl)phenyl)-1-phenylprop-2-en-1-one (**3a**).

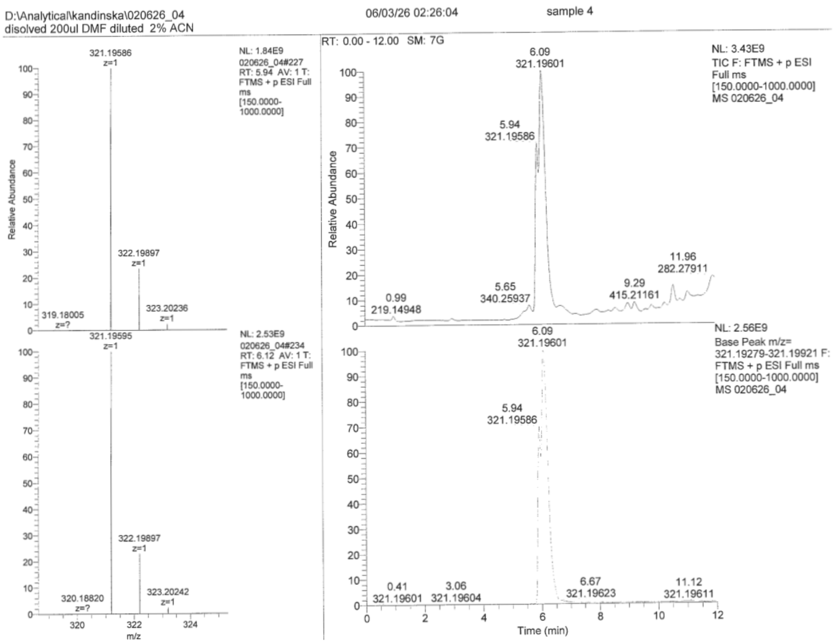

Figure S4. ^{1}H-NMR Spectrum ($CDCl_3$, 500 MHz) of (*E*)-1-(4-(Dimethylamino)phenyl)-3-(4-(4-ethylpiperazin-1-yl)phenyl)prop-2-en-1-one (**3b**).

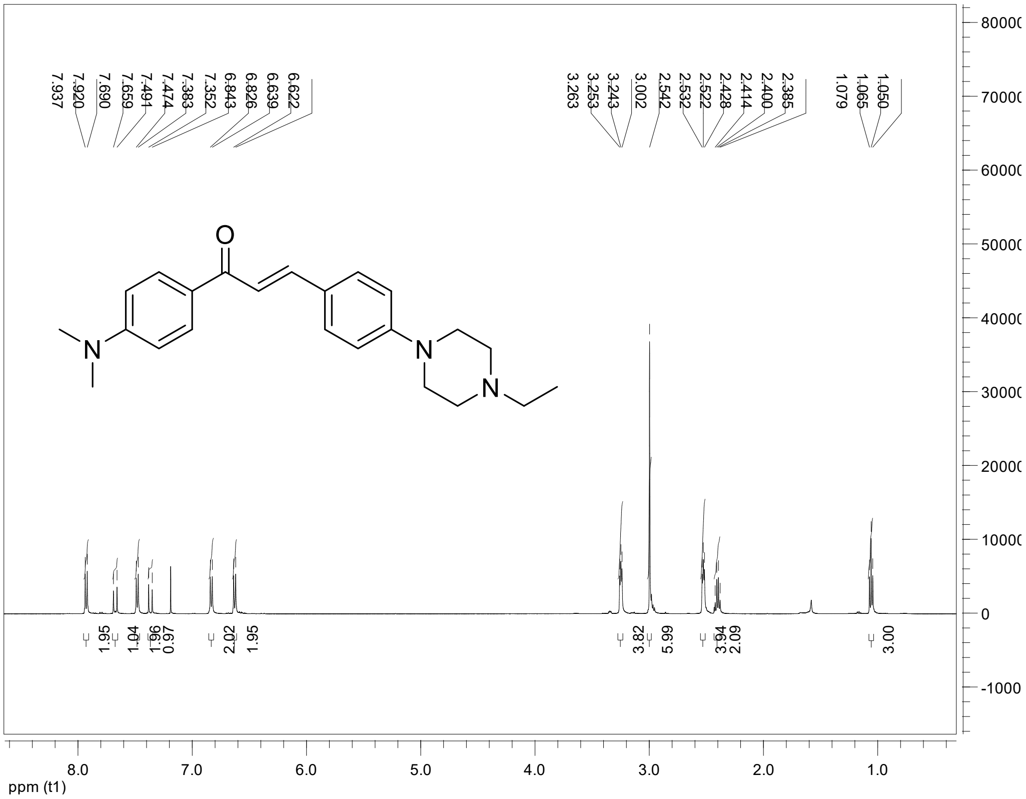

Figure S5. ^{13}C-NMR Spectrum ($CDCl_3$, 125.76 MHz) of (*E*)-1-(4-(Dimethylamino)phenyl)-3-(4-(4-ethylpiperazin-1-yl)phenyl)prop-2-en-1-one (**3b**).

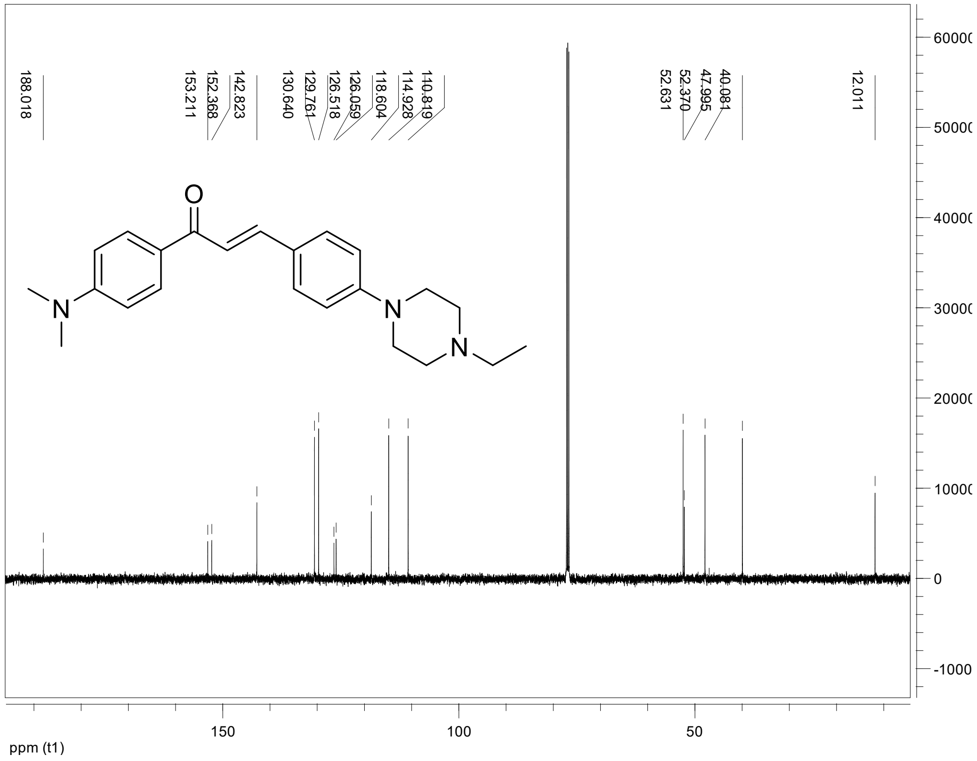

Figure S6. LC-MS of (*E*)-1-(4-(Dimethylamino)phenyl)-3-(4-(4-ethylpiperazin-1-yl)phenyl)prop-2-en-1-one (**3b**).

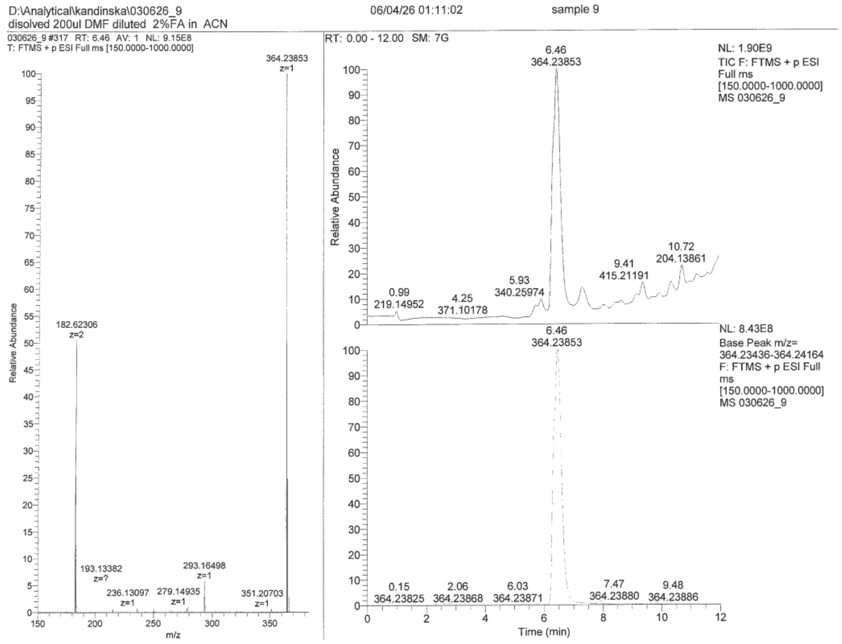

Figure S7. ^{1}H-NMR Spectrum ($CDCl_3$, 500 MHz) of (*E*)-3-(4-(4-Ethylpiperazin-1-yl)phenyl)-1-(4-methoxyphenyl)prop-2-en-1-one (**3c**).

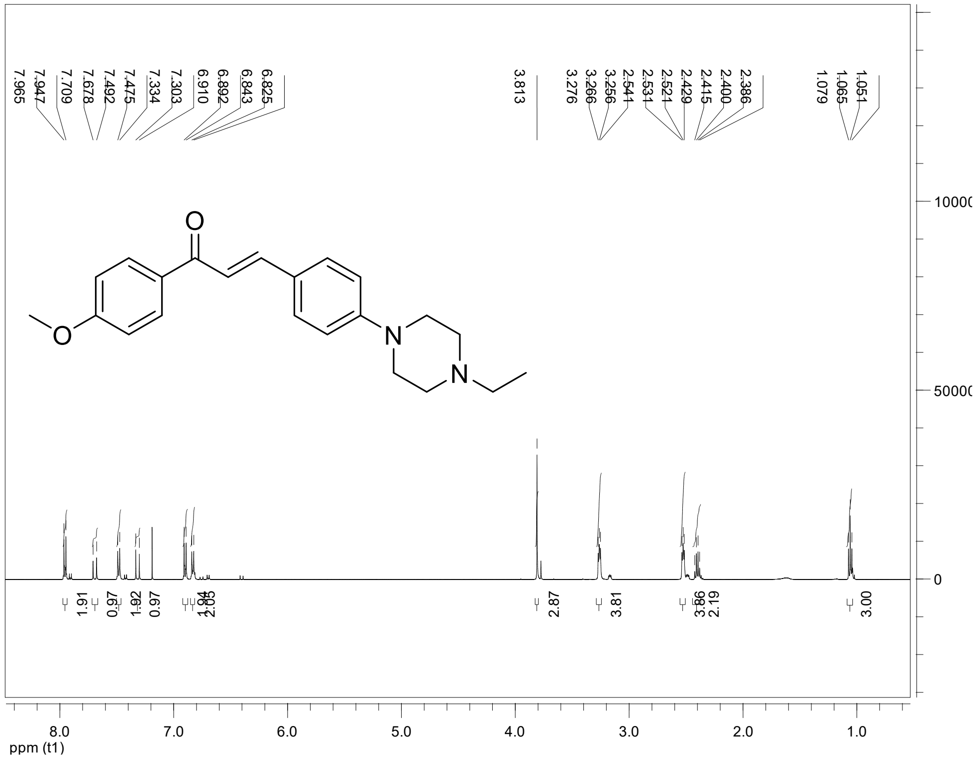

Figure S8. ^{13}C-NMR Spectrum ($CDCl_3$, 125.76 MHz) of (*E*)-3-(4-(4-Ethylpiperazin-1-yl)phenyl)-1-(4-methoxyphenyl)prop-2-en-1-one (**3c**).

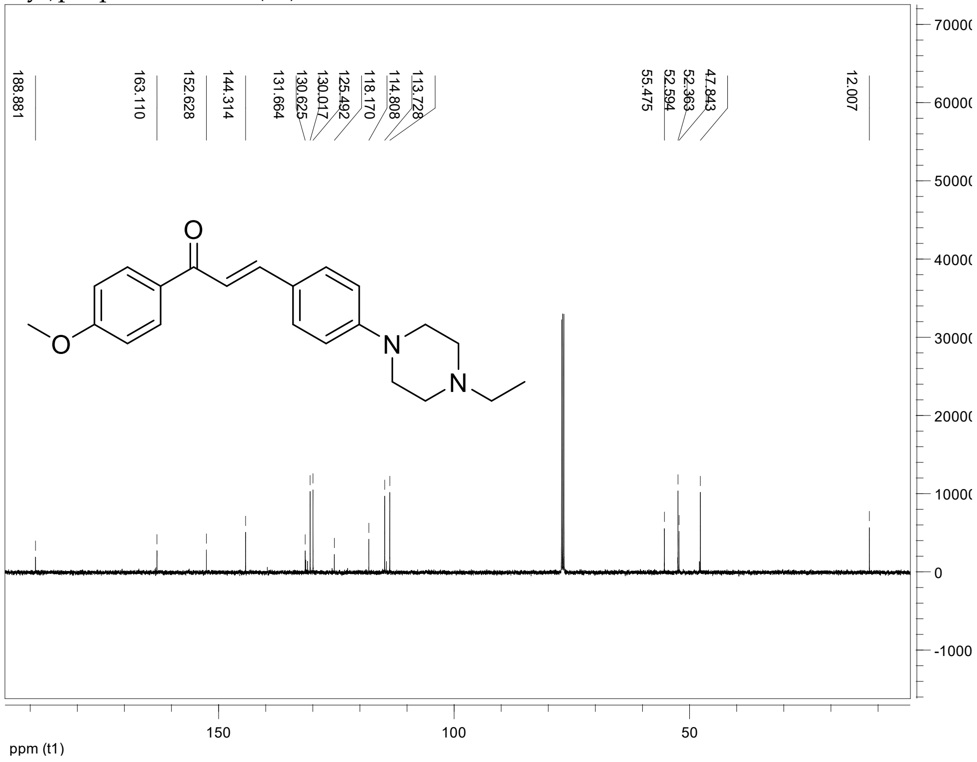

Figure S9. LC-MS of (*E*)-3-(4-(4-Ethylpiperazin-1-yl)phenyl)-1-(4-methoxyphenyl)prop-2-en-1-one (**3c**).

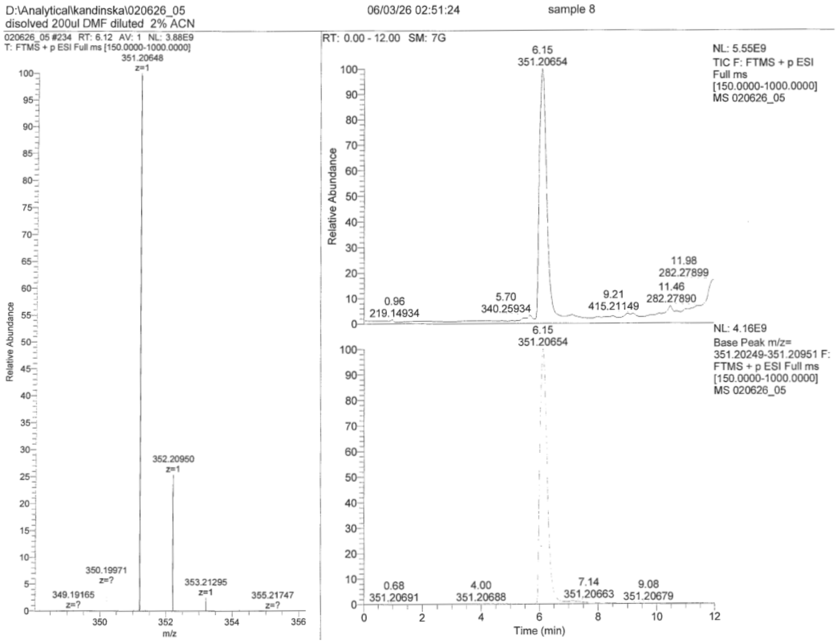

Figure S10. ^{1}H-NMR Spectrum ($CDCl_3$, 500 MHz) of 1-(4-(1,3-Diphenyl-4,5-dihydro-1*H*-pyrazol-5-yl)phenyl)-4-ethylpiperazine (**4a**).

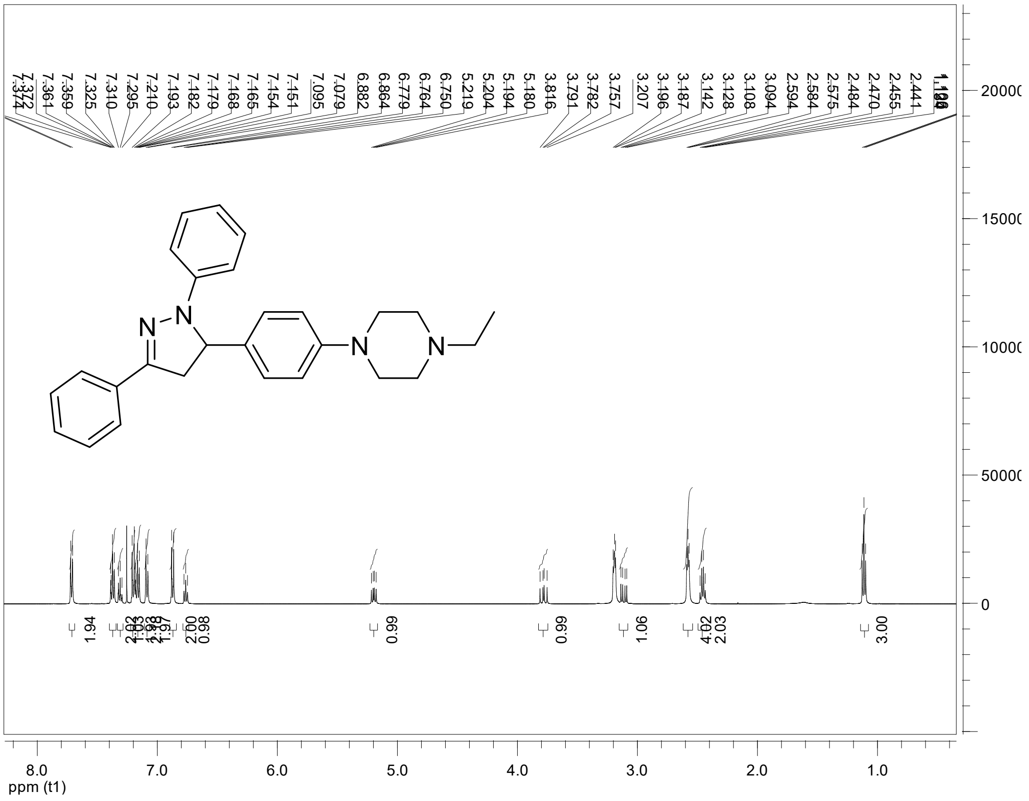

Figure S11. ^{13}C-NMR Spectrum ($CDCl_3$, 125.76 MHz) of 1-(4-(1,3-Diphenyl-4,5-dihydro-1*H*-pyrazol-5-yl)phenyl)-4-ethylpiperazine (**4a**).

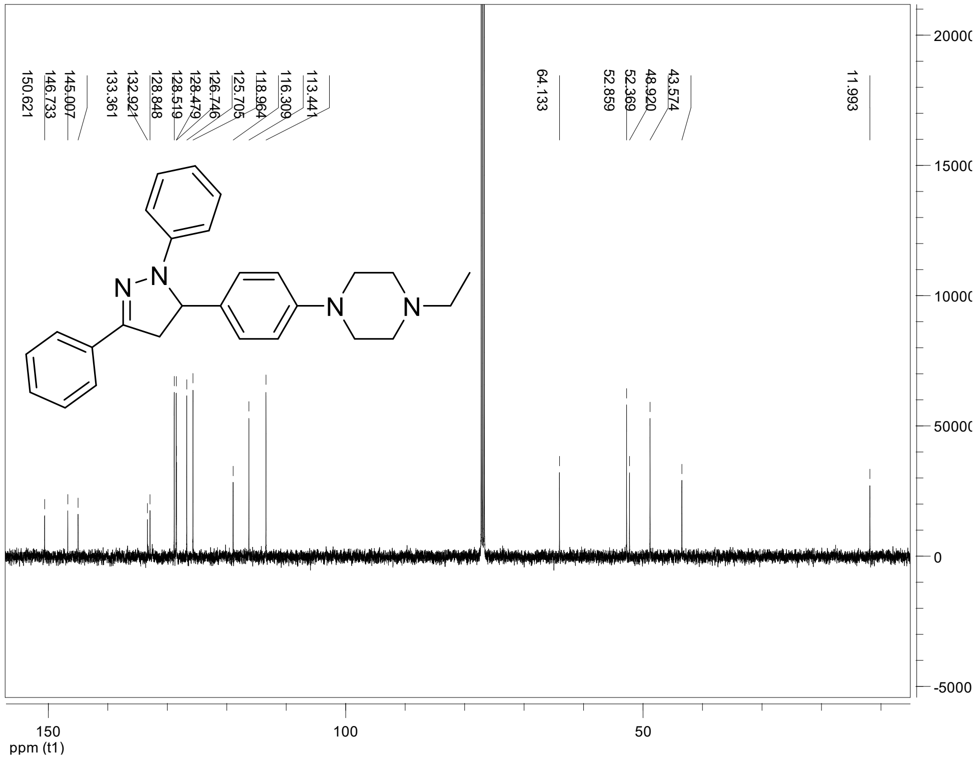

Figure S12. LC-MS of 1-(4-(1,3-Diphenyl-4,5-dihydro-1*H*-pyrazol-5-yl)phenyl)-4-ethylpiperazine (**4a**).

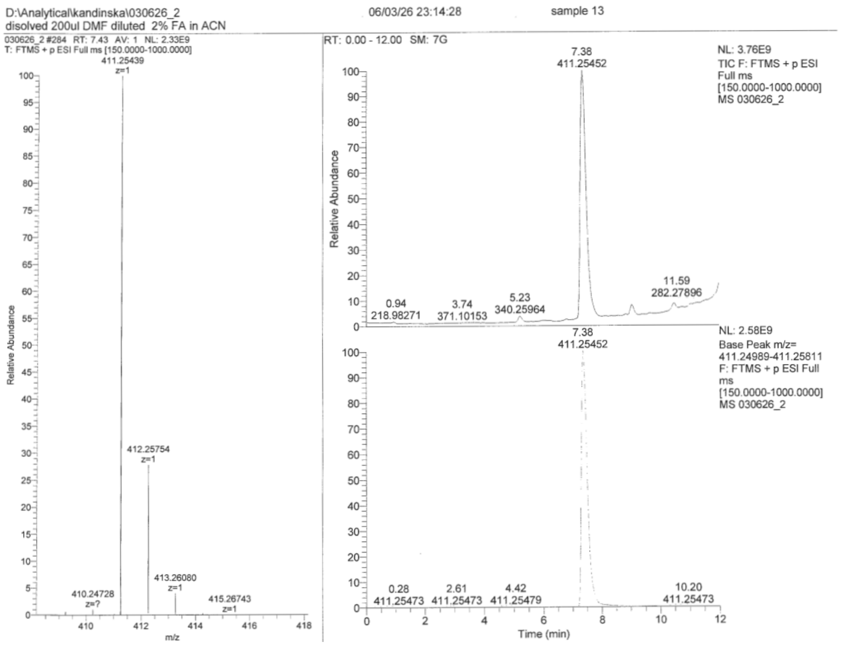

Figure S13. ^{1}H-NMR Spectrum ($CDCl_3$, 500 MHz) of 4-(5-(4-(4-Ethylpiperazin-1-yl)phenyl)-1-phenyl-4,5-dihydro-1*H*-pyrazol-3-yl)-*N*,*N*-dimethylaniline (**4b**).

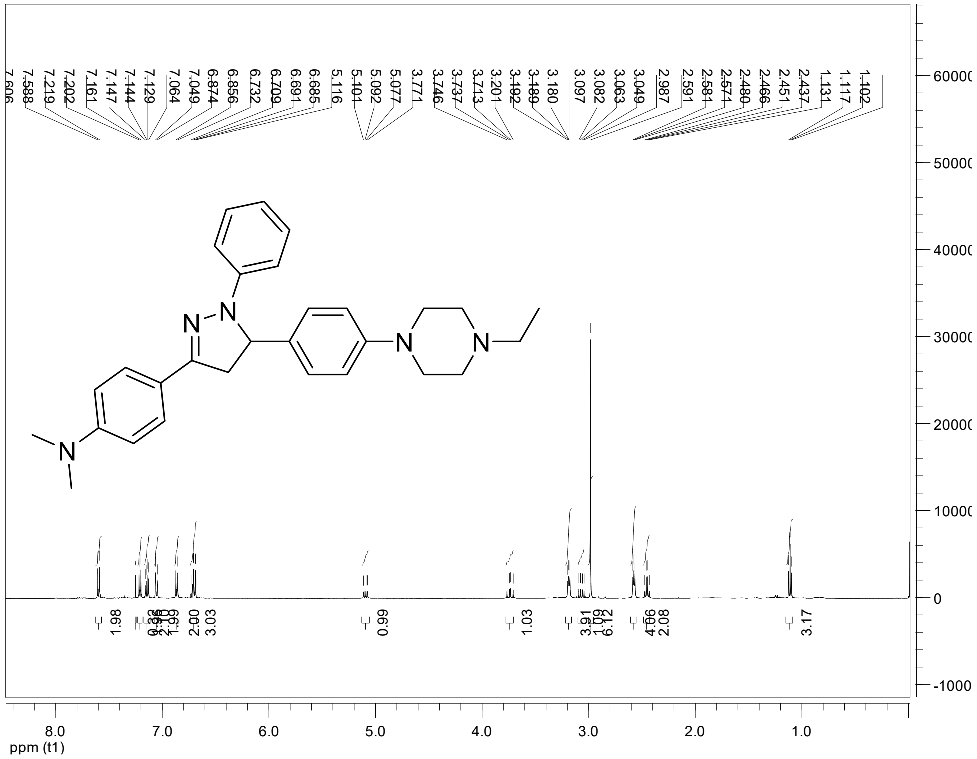

Figure S14. ^{13}C-NMR Spectrum ($CDCl_3$, 125.76 MHz) of 4-(5-(4-(4-Ethylpiperazin-1-yl)phenyl)-1-phenyl-4,5-dihydro-1*H*-pyrazol-3-yl)-*N*,*N*-dimethylaniline (**4b**).

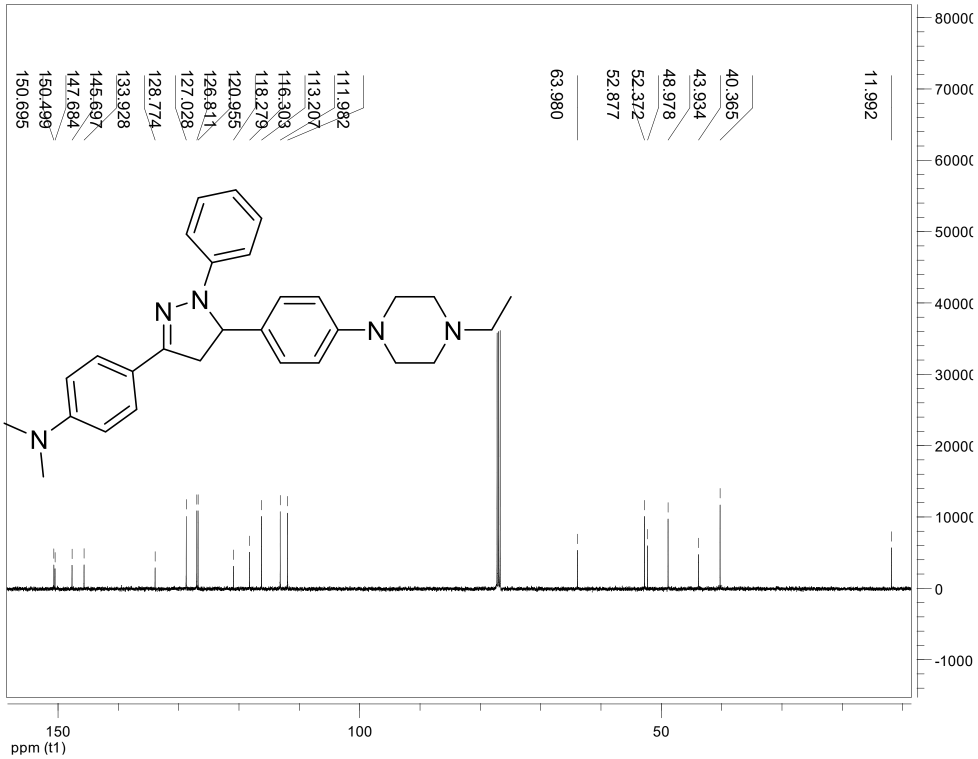

Figure S15. LC-MS of 4-(5-(4-(4-Ethylpiperazin-1-yl)phenyl)-1-phenyl-4,5-dihydro-1*H*-pyrazol-3-yl)-*N*,*N*-dimethylaniline (**4b**).

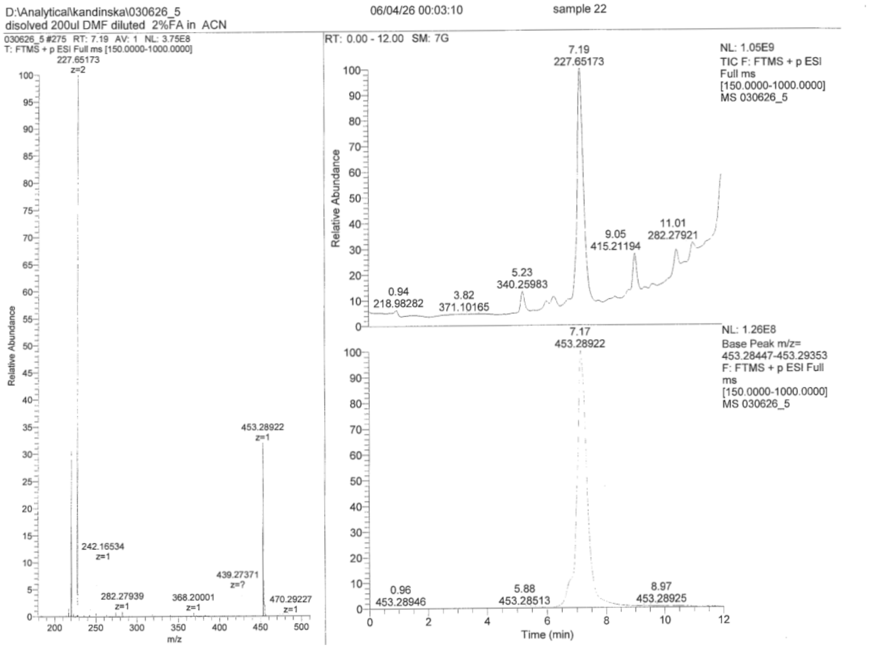

Figure S16. ^{1}H-NMR Spectrum ($CDCl_3$, 500 MHz) of 1-Ethyl-4-(4-(3-(4-methoxyphenyl)-1-phenyl-4,5-dihydro-1*H*-pyrazol-5-yl)phenyl)piperazine (**4c**).

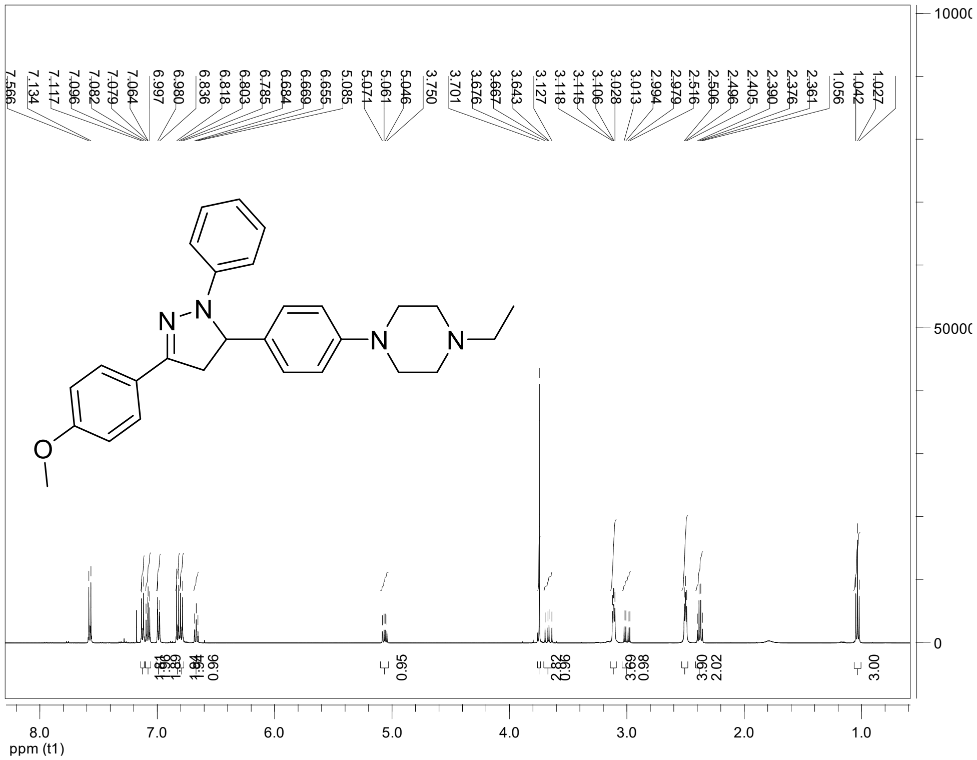

Figure S17. ^{13}C-NMR Spectrum ($CDCl_3$, 125.76 MHz) of 1-Ethyl-4-(4-(3-(4-methoxyphenyl)-1-phenyl-4,5-dihydro-1*H*-pyrazol-5-yl)phenyl)piperazine (**4c**).

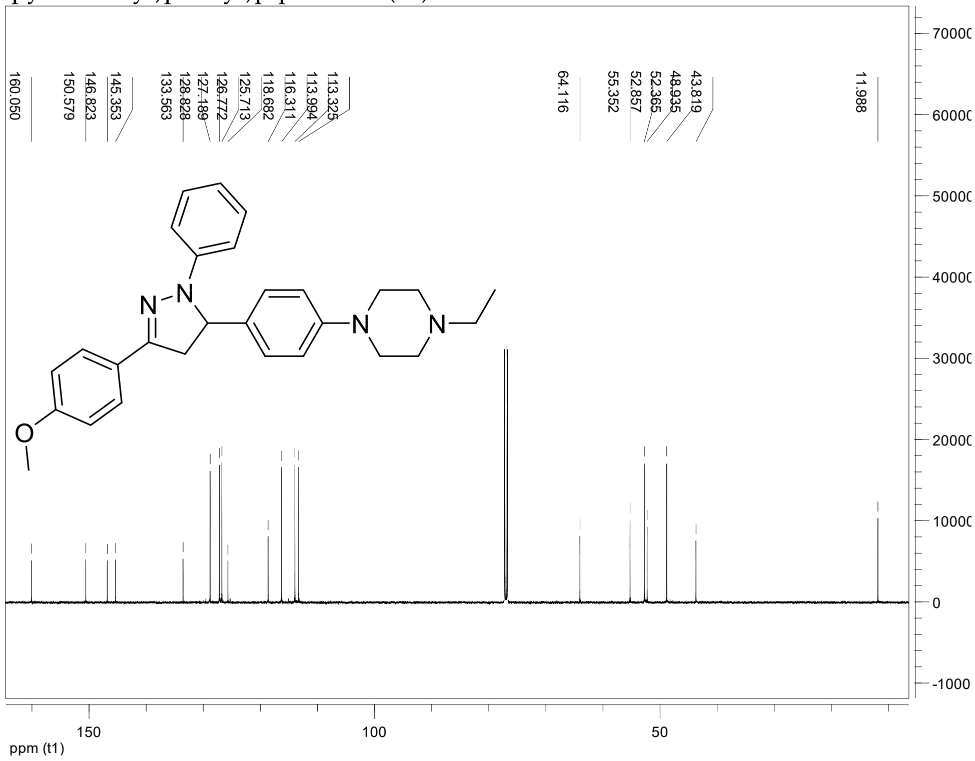

Figure S18. LC-MS of 1-Ethyl-4-(4-(3-(4-methoxyphenyl)-1-phenyl-4,5-dihydro-1*H*-pyrazol-5-yl)phenyl)piperazine (**4c**).

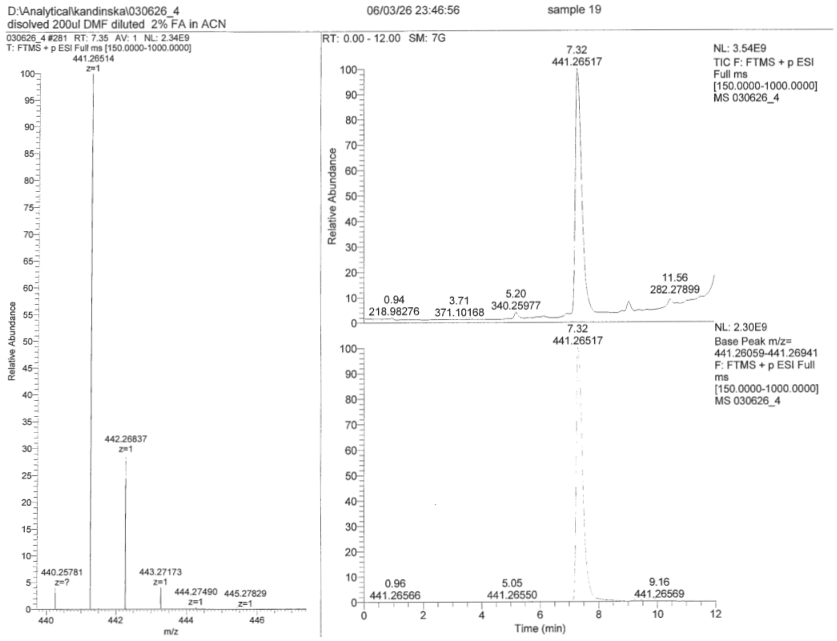

Figure S19. ^{1}H-NMR Spectrum ($CDCl_3$, 500 MHz) of 1-(4-(1,3-Diphenyl-4,5-dihydro-1*H*-pyrazol-5-yl)phenyl)piperidine (**4d**).

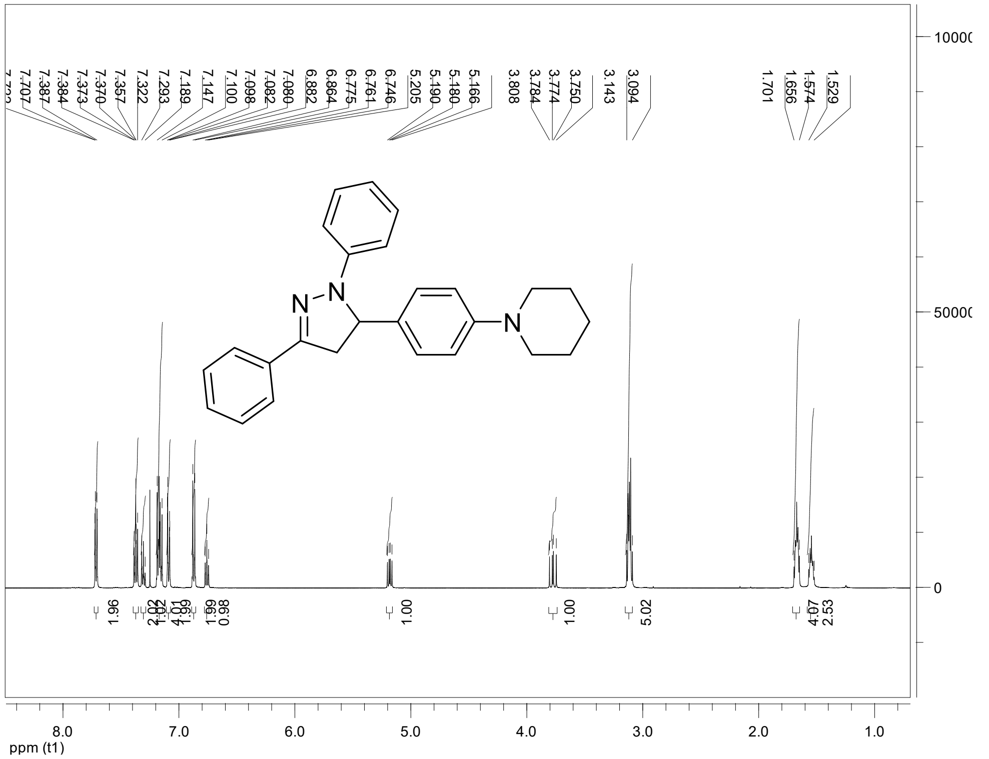

Figure S20. ^{13}C-NMR Spectrum ($CDCl_3$, 125.76 MHz) of of 1-(4-(1,3-Diphenyl-4,5-dihydro-1*H*-pyrazol-5-yl)phenyl)piperidine (**4d**).

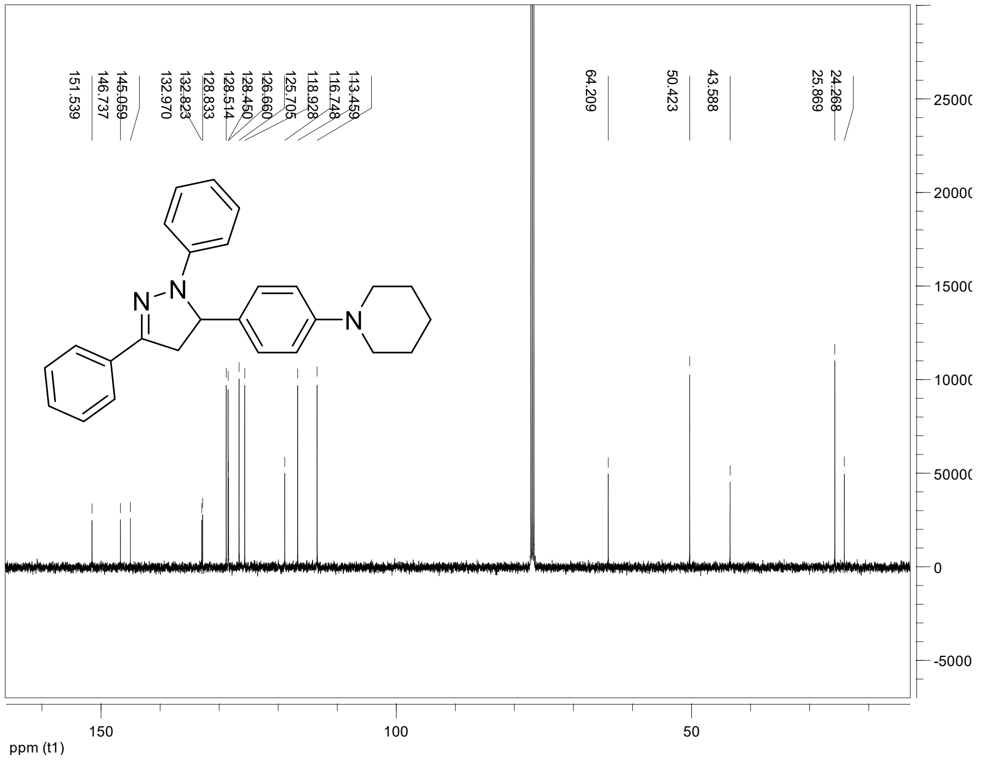

Figure S21. LC-MS of of 1-(4-(1,3-Diphenyl-4,5-dihydro-1*H*-pyrazol-5-yl)phenyl)piperidine (**4d**).

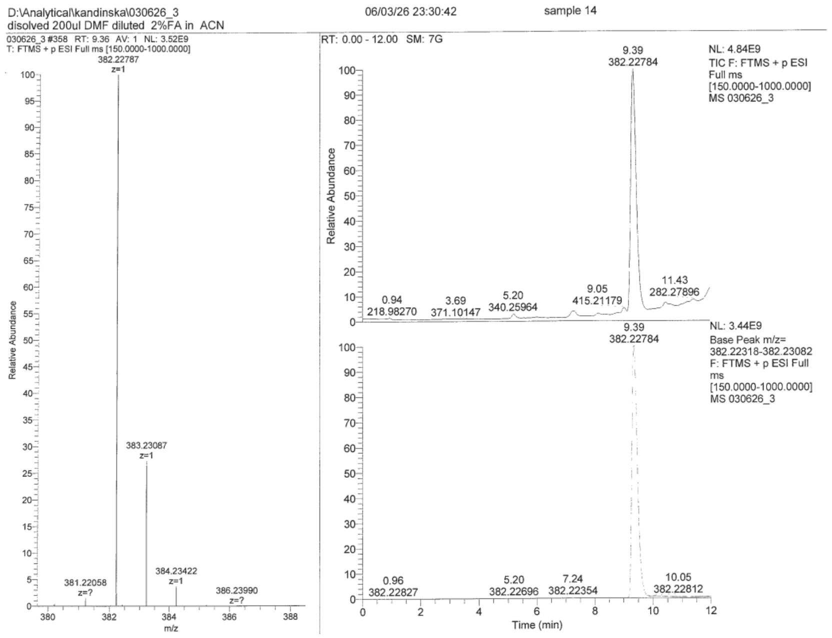

Supplement: Supplementary file 1 [file molecules-31-02380-s001.zip › molecules-4393016-supplementary.pdf]
